# Targeting BET Proteins Decreases Hyaluronidase-1 in Pancreatic Cancer

**DOI:** 10.3390/cells12111490

**Published:** 2023-05-27

**Authors:** Krishan Kumar, Deepak Kanojia, David J. Bentrem, Rosa F. Hwang, Jonathan P. Butchar, Susheela Tridandapani, Hidayatullah G. Munshi

**Affiliations:** 1 Department of Internal Medicine, Division of Hematology, and Arthur G. James Comprehensive Cancer Center, The Ohio State University College of Medicine, Columbus, OH 43210, USA; 2 Department of Medicine, Feinberg School of Medicine, Northwestern University, Chicago, IL 60611, USA; 3 Robert H. Lurie Comprehensive Cancer Center, Northwestern University, Chicago, IL 60611, USA; 4 Department of Neurological Surgery, Feinberg School of Medicine, Northwestern University, Chicago, IL 60611, USA; 5 Department of Surgery, Feinberg School of Medicine, Northwestern University, Chicago, IL 60611, USA; 6 Jesse Brown VA Medical Center, Chicago, IL 60612, USA; 7 Department of Surgical Oncology, The University of Texas MD Anderson Cancer Center, Houston, TX 77030, USA

**Keywords:** bromodomain and extra-terminal (BET) proteins, BET inhibitors, BRD2, hyaluronidases, HYAL1, pancreatic cancer, apoptosis, proliferation

## Abstract

Background: Pancreatic ductal adenocarcinoma (PDAC) is characterized by the presence of dense stroma that is enriched in hyaluronan (HA), with increased HA levels associated with more aggressive disease. Increased levels of the HA-degrading enzymes hyaluronidases (HYALs) are also associated with tumor progression. In this study, we evaluate the regulation of HYALs in PDAC. Methods: Using siRNA and small molecule inhibitors, we evaluated the regulation of HYALs using quantitative real-time PCR (qRT-PCR), Western blot analysis, and ELISA. The binding of BRD2 protein on the HYAL1 promoter was evaluated by chromatin immunoprecipitation (ChIP) assay. Proliferation was evaluated by WST-1 assay. Mice with xenograft tumors were treated with BET inhibitors. The expression of HYALs in tumors was analyzed by immunohistochemistry and by qRT-PCR. Results: We show that HYAL1, HYAL2, and HYAL3 are expressed in PDAC tumors and in PDAC and pancreatic stellate cell lines. We demonstrate that inhibitors targeting bromodomain and extra-terminal domain (BET) proteins, which are readers of histone acetylation marks, primarily decrease HYAL1 expression. We show that the BET family protein BRD2 regulates HYAL1 expression by binding to its promoter region and that HYAL1 downregulation decreases proliferation and enhances apoptosis of PDAC and stellate cell lines. Notably, BET inhibitors decrease the levels of HYAL1 expression in vivo without affecting the levels of HYAL2 or HYAL3. Conclusions: Our results demonstrate the pro-tumorigenic role of HYAL1 and identify the role of BRD2 in the regulation of HYAL1 in PDAC. Overall, these data enhance our understanding of the role and regulation of HYAL1 and provide the rationale for targeting HYAL1 in PDAC.

## 1. Background

Pancreatic ductal adenocarcinoma (PDAC), one of the most lethal malignancies, is projected to become the second leading cause of cancer-related death by 2030 [1]. PDAC is associated with a pronounced stroma that can account for up to 90% of tumor mass [2,3]. The PDAC stroma, which is enriched in collagen and hyaluronan (HA), can contribute to tumor progression and treatment resistance [4]. Increased HA accumulation is associated with more aggressive disease and therapy resistance in human tumors [5,6,7].

HA is a linear, soluble glycosaminoglycan (GAG) macromolecule consisting of repeated chains of disaccharides N-acetyl glucosamine and D-glucuronic acid [8]. In normal physiological conditions, HA levels are controlled by a balance between HA production and degradation. HA is synthesized by HA synthases (HASs) and degraded by HA-degrading enzymes hyaluronidases (HYALs). HA performs diverse functions depending on its size. The high molecular weight HA (HMW-HA) promotes anti-inflammatory, anti-proliferative and anti-angiogenic effects, while the low molecular weight HA (LMW-HA) is known to promote cell proliferation, inflammation, and angiogenesis [9,10].

HA molecular weight depends on the activity of HYALs; HYAL2 cleaves HMW-HA fragments which are further cleaved into LMW-HA fragments by HYAL1 [11,12]. HYALs are also increased in malignant tumors and are associated with cancer progression, invasion, and metastasis [13,14]. Of the six human HYAL-related genes, HYAL1, HYAL2, and HYAL3 are widely expressed and play key roles in HA degradation [15,16]. HYAL1 overexpression is also associated with PDAC progression [17,18]. However, little is known about the regulation and functional role of HYALs in PDAC.

Bromodomain and extra terminal (BET) proteins, which include BRD2, BRD3, BRD4, and the testes-specific BRDT, regulate the transcription of genes involved in several human diseases [19,20,21,22]. BET proteins bind to acetylation marks on histones and enhance the recruitment of transcription factors and other chromatin regulators during transcription [21,22]. The BET inhibitor JQ1 suppresses cancer cell migration, colony formation, invasion, and cell cycle progression and promotes cellular differentiation, DNA damage response, and apoptosis with minimal effect on normal healthy cells [23,24,25,26]. We previously showed that BET inhibitors could attenuate stroma and decrease collagen production by pancreatic stellate cells [27], the key regulators of fibrosis in vivo [27]. Significantly, we showed that the BET proteins BRD2, BRD3, and BRD4 differentially regulate collagen production by pancreatic stellate cells [27]. While the role of BET proteins in regulating collagen expression is now well established [28], the role of BET proteins in regulating HA and HYAL expression has not been previously studied.

In this report, we show that HYAL1, HYAL2, and HYAL3 are expressed in human PDAC tumors, as well as in stellate and PDAC cell lines. Therapeutic and genetic inhibition of BET proteins specifically downregulates HYAL1 expression, and HYAL1 downregulation is most consistently seen with BRD2 silencing. We show that the BRD2 protein directly binds to the HYAL1 promoter. We also show that HYAL1 downregulation decreases proliferation and enhances apoptosis. BET inhibitors also decrease the levels of HYAL1 expression in vivo but do not affect the levels of HYAL2 or HYAL3. Together, our results demonstrate the pro-tumorigenic role of HYAL1 in pancreatic cancer and identify the role of BRD2 in the regulation of HYAL1 in PDAC.

## 2. Methods

Pancreatic cancer samples—Primary tumor specimens were obtained from PDAC patients who underwent surgical resection at Northwestern Memorial Hospital in Chicago between 2015 and 2016 on an IRB-approved protocol. Anonymized tissue specimens were snap-frozen in RNAlater (QIAGEN GmbH, Hilden, Germany) for RNA extraction. Human PDAC tissue microarrays (TMAs) were purchased from Reveal Biosciences, Inc. (San Diego, CA, USA).

Cell lines, antibodies and reagents—PDAC cancer cell lines Panc1, FG, AsPC1, MiaPaCa2, and Panc02.03 were obtained from American Type Culture Collection (ATCC, Manassas, VA, USA). All cell lines were screened periodically for mycoplasma contamination, and cell lines were also profiled by short tandem repeat (STR) analysis and authenticated using published reference databases. The human pancreatic stellate cell line (Stl), immortalized with telomerase and SV40 large T antigen and characterized, was obtained from Rosa F. Hwang (MD Anderson Cancer Center, Houston, TX, USA) [29,30]. All cell lines were grown in DMEM supplemented with 10% fetal bovine serum (FBS) plus antibiotics (100 U/mL Penicillin and 100 μg/mL Streptomycin) in a humidified incubator at 37 °C and 5% CO_2_. HPDE cells were cultured in keratinocyte serum-free (KSF) media supplemented with 0.1 mg/mL Bovine Pituitary Extract, 0.01 µg/mL Epidermal Growth Factor, 100 units/mL of penicillin, and 100 μg/mL of streptomycin. To avoid the effects of media variations on mRNA expression, once HPDE cells reached ~70% confluency, KSF media was replaced with DMEM supplemented with 10% FBS for 48 h prior to RNA extraction.

Antibodies against BRD2, BRD3, and BRD4 were purchased from Abcam, Waltham, MA. Antibodies against cleaved caspase-3 (Asp175) and cleaved PARP (Asp214) were obtained from Cell Signaling Technologies, Danvers, MA, USA. GAPDH antibody and biotinylated Hyaluronic Acid Binding Protein (HABP) were from Millipore Sigma (St. Louis, MO, USA). HYAL1 antibody was obtained from GeneTex Inc., Irvine, CA, USA, and the Ki67 antibody was from Invitrogen, CA, USA. Secondary anti-mouse IgG and anti-rabbit IgG antibodies were purchased from Sigma. The BET inhibitor JQ1 was obtained from Tocris Bioscience, Minneapolis, MN, USA. Silencer^®^ Select siRNAs against BRD2, BRD3, BRD4 and Silencer Negative Control siRNA were purchased from Ambion, Thermo Fisher Scientific. ON-TARGETplus siRNAs against HYAL1, HYAL2 and HYAL3 were purchased from Dharmacon, Horizon Discovery. Additional Silencer^®^ Select siRNAs against HYAL1 were obtained from Ambion. The Human Hyaluronidase 1/HYAL1 DuoSet ELISA kit was purchased from R&D Systems, Minneapolis, MN, USA.

Cell proliferation assay—Approximately 5000 cells were seeded in 96-well plates. After 3 days of JQ1 treatment or after 4 days of transfection with siRNAs against HYAL1, HYAL2 or HYAL3, Cell Proliferation Reagent WST-1 reagent (Hoffmann-La Roche Inc./Sigma, Mannheim, Germany) was added into the media at a 1:100 dilution factor. Absorbance was measured at 490 nm using a microplate reader (EPOCH2 microplate reader, BioTek, Winooski, VT, USA), according to the manufacturer’s instructions as described previously [31].

Western blot analysis—Western blotting was performed using standard protocols. Whole-cell lysates were prepared with RIPA lysis buffer supplemented with phosphatase (Phosphatase Inhibitor Cocktail Set III, EMD Millipore) and protease (Protease Inhibitor Cocktail Set III, EMD Millipore) inhibitors. Protein concentrations were determined using Precision Red Advanced Protein Assay ADV02 from Cytoskeleton, Inc. (Denver, CO, USA). Cell lysates were denatured in Pierce 2X Lane Marker Reducing Sample Buffer from Thermo Scientific at 100 °C for 5 min and loaded onto 8–12% SDS-PAGE. After electrophoresis, the proteins were transferred to an Immobilon-P PVDF membrane (Millipore, Burlington, MA, USA) using the Trans-Blot Turbo transfer system (Bio-Rad, Hercules, CA, USA). The membranes were blocked with 5% BSA-TBST for 1 h at room temperature and overnight incubated with primary antibodies at 4 °C. Primary antibodies were detected with anti-mouse horseradish peroxidase (HRP)-conjugated antibody (Sigma) or anti-rabbit HRP-conjugated antibody (Sigma) followed by enhanced chemiluminescence using Pierce ECL Western Blotting Substrate (Thermo Scientific, Waltham, MA, USA). Chemiluminescence was detected using autoradiography films. Immunoblotting for BRD2, BRD3, BRD4, cleaved caspase-3, cleaved PARP and GAPDH were done as described previously [32,33].

Histochemistry—TMAs were trichrome-stained to assess for fibrosis as described previously [30]. TMAs were also stained with biotinylated HABP to assess for hyaluronan. TMAs were scanned by using Hamamatsu 2.0 HT NanoZoomer (Hamamatsu Photonics, Hamamatsu City, Japan), a Digital Imaging system at 20X magnification.

Immunohistochemistry—Mouse tumor specimens were stained for Ki67 and HYAL1 using Dako Autostainer Plus (Dako, Inc., Carpinteria, CA, USA). Slides were scanned by using Hamamatsu 2.0 HT NanoZoomer, a Digital Imaging system at magnification 20× for further analysis. Proliferation marker Ki67 positive cells per field were quantified by ImageJ analysis as previously described. Expression of HYAL1 was graded, and a score of less than 25% (1+), 25% to 75% (2+), or more than 75% (3+) as previously described [27,31].

Transient transfection with siRNA—Cells were seeded in a 6-well plate for protein lysates or a 12-well plate for mRNA extraction. Cells were allowed to adhere overnight. The next day transfections were carried out using Lipofectamine RNAimax (Invitrogen, Waltham, MA, USA) and Opti-MEM I Reduced-Serum Medium (Thermo Scientific) according to the manufacturer’s instructions, as detailed in previous studies [27,34].

Quantitative real-time PCR—Cells were lysed in RLT buffer (Qiagen, Hilden, Germany). Primary human PDAC tumors and tumors from nude mice bearing PDAC cells were homogenized in RLT buffer using TissueRuptor (Qiagen). Total RNA was isolated using RNeasy Mini Kit (QIAGEN GmbH, Hilden, Germany) according to the manufacturer’s protocol. RNA concentration and purity were measured using a NanoDrop OneC spectrophotometer (Thermo Scientific, Waltham, MA, USA). First-strand cDNA was synthesized from 1.5 μg of total RNA using a high-capacity cDNA transcription kit (Applied Biosystems, Waltham, MA, USA). Quantitative gene expression was performed with gene-specific TaqMan primers, TaqMan Universal PCR Master Mix, and the 7900HT Fast Real-Time PCR System (Applied Biosystems, Foster City, CA, USA). The relative mRNA expression levels were calculated using the 2^−ΔΔCT^ method.

Chromatin immunoprecipitation (ChIP) assay—ChIP analysis was performed using a Millipore Sigma kit according to the manufacturer’s instructions. Ten million AsPC1 cells were seeded in 150 mm culture dishes and allowed to adhere overnight. The next day, cells were treated with JQ1 or DMSO overnight. The adherent cells were fixed and crosslinked in 1% formaldehyde at room temperature (RT) for 10 min. The unreacted formaldehyde was then neutralized with 125 mM glycine for 5 min. Cells were washed twice with ice-cold PBS. The cells were scraped using a sterile cell scraper in 2 mL of ice-cold PBS supplemented with Protease Inhibitor Cocktail II. After centrifugation at 800× *g* at 4 °C for 5 min, the cell pellet was resuspended in 800 µL ice-cold EZ-Zyme Lysis Buffer containing Protease Inhibitor Cocktail II. Chromatin fragments were prepared using the EZ-Zyme Chromatin Prep kit (17–375, Millipore), and ChIP performed using the EZ-Magna ChIP A/G Chromatin Immunoprecipitation kit (17–10086, Millipore) and anti-BRD2 antibody (A302-583A, Bethyl Laboratories), or control IgG antibody (2729, Cell Signaling). DNA was purified after reverse crosslinking. Purified DNA was then analyzed by PCR using KiCqStart SYBR Green qPCR ReadyMix (KCQS02, Sigma) and primers specific for the HYAL1 promoter: forward 5′-AACCAAGATCCCTTTGCCAG-3′ and reverse 5′-TCCAAATTTCCTGACCCCAG-3′ [35].

HYAL1 ELISA—Measurements of HYAL1 Concentrations in tissue culture media of the cell-free supernatants were determined using a Human Hyaluronidase 1/HYAL1 DuoSet ELISA kit (R&D Systems, Inc., Minneapolis, MI, USA) according to manufacturer’s instructions with slight modification. For low HYAL1 expressing cells, Panc1 and stellate tissue culture media supernatants were concentrated two times using Amicon Ultra-2 mL Centrifugal filters Ultracel-10K (Millipore). Quantification was performed by measuring the absorbance at 450 nm with 570 as a reference using a microplate reader. A curve of absorbance versus concentration of HYAL1 in the standard wells was determined by interpolation from a standard curve.

In vivo study—All animal studies were completed per NIH guidelines on the care and use of laboratory animals for research purposes. The protocol was approved by the Institutional Animal Care and Use Committee (IACUC) of Northwestern University (Chicago, IL, USA). Six- to eight-week-old athymic nude female mice were obtained from Charles River and maintained in a specific pathogen-free facility. Five million Panc1 cells were implanted subcutaneously in the flank of nude mice as 100 μL cell suspensions with an equal volume of Matrigel (BD Biosciences, Franklin Lakes, NJ, USA) [36]. Tumors were measured twice a week with a digital caliper, and volumes (V) were calculated using the formula V = ½ Length × Width^2^. Once the tumor volume reached approximately 200 mm^3^, mice were randomized into two treatment groups: control (DMSO) and JQ1 (50 mg/kg). Treatments were administered by intraperitoneal (i.p.) injections five days per week (Monday–Friday) for three weeks in a suspension containing 10% hydroxypropyl-β-cyclodextrin in double-distilled water. At the end of the study, mice were euthanized by CO_2_ inhalation and cervical dislocation, and the tumors were excised, weighed, and photographed. Tumor fragments were either processed by formalin fixation before paraffin embedding for IHC or frozen for later RNA extraction. The number of Ki67+ cells in each section was calculated by ImageJ. At least four different sections were taken for each tumor.

Statistical analysis—In vivo and in vitro results were compared using a two-tailed *t*-test analysis or Mann–Whitney U test. Error bars represent SD or SEM as specified. All statistical analyses were done using Microsoft Excel and GraphPad Prism (GraphPad Software Inc., San Diego, CA, USA). A *p*-value of less than 0.05 was considered significant.

## 3. Results

**Figure 1:**
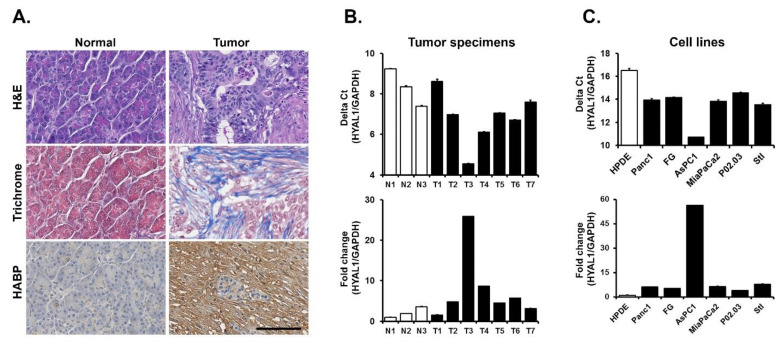
Expression of Hyaluronidase-1 (HYAL1) in PDAC specimens and cell lines. (**A**) Normal human pancreatic tissue and PDAC tumor sections were stained with H&E, trichrome stained to evaluate for fibrosis (blue), and stained with hyaluronan binding protein (HAPB). Scale bar = 100 μm. (**B**) Hyaluronidase-1 (HYAL1) mRNA expression level was analyzed in three normal human pancreatic tissue (N1-N3) and seven PDAC specimens (T1-T7). The relative HYAL1 mRNA expression was normalized to HYAL1 mRNA levels present in normal human pancreatic tissue 1 (N1) for tumor specimens, delta Ct (upper panel) and fold change (lower panel). Mean ± SD. (**C**) Hyaluronidase-1 (HYAL1) mRNA expression levels were analyzed in immortalized human pancreatic duct epithelial (HPDE) cells, in a panel of five human PDAC cell lines, and in an immortalized human pancreatic stellate cell line (Stl). The relative HYAL1 mRNA expression was normalized to HYAL1 mRNA levels present in HPDE cells, with results shown as delta Ct (upper panel) and fold change (lower panel). Mean ± SD. The gene expression results are representative of three independent experiments.

Expression of Hyaluronidase-1 (HYAL1) in PDAC specimens and cell lines. Human PDAC tumors have increased fibrosis, as demonstrated by trichrome staining, and increased hyaluronan (HA) expression, as demonstrated by increased staining for HA binding protein (HABP) (Figure 1A). Since HA undergoes dynamic regulation by HYALs [37,38,39], we evaluated the expression of HYAL1, HYAL2, and HYAL3 by RT-qPCR in three normal pancreatic tissue samples and seven PDAC tumor specimens. Variable levels of HYAL1 expression were seen in all tumor specimens, with the highest HYAL1 expression observed in PDAC specimen #3. There was ~25-fold higher expression of HYAL1 in PDAC specimen #3 compared to the adjacent normal pancreatic tissue sample #1 (Figure 1B). Overall, there was increased expression of HYAL1 in human PDAC tumors compared to adjacent normal pancreatic tissue samples. In contrast, there was not a significant difference in HYAL2 and HYAL3 expression between human PDAC tumors and adjacent normal tissue (Supplementary File: Figure S1A,C).

Similarly, we evaluated HYAL1, HYAL2, and HYAL3 mRNA expression in immortalized human pancreatic duct epithelial (HPDE) cells, in five PDAC cell lines, and in an immortalized pancreatic stellate cell line. Varying levels of HYAL1 expression were seen in PDAC cell lines, with the AsPC1 cell line exhibiting the highest HYAL1 mRNA expression. There was over 50-fold higher HYAL1 mRNA in the AsPC1 cell line compared to HPDE cells (Figure 1C). Overall, there was increased HYAL1 expression in human PDAC cell lines compared to HPDE cells. In contrast, there was minimal to no difference in the expression of HYAL2 and HYAL3 expression between HPDE cells and the five pancreatic cancer cell lines (Supplementary File: Figure S1B,D).

**Figure 2:**
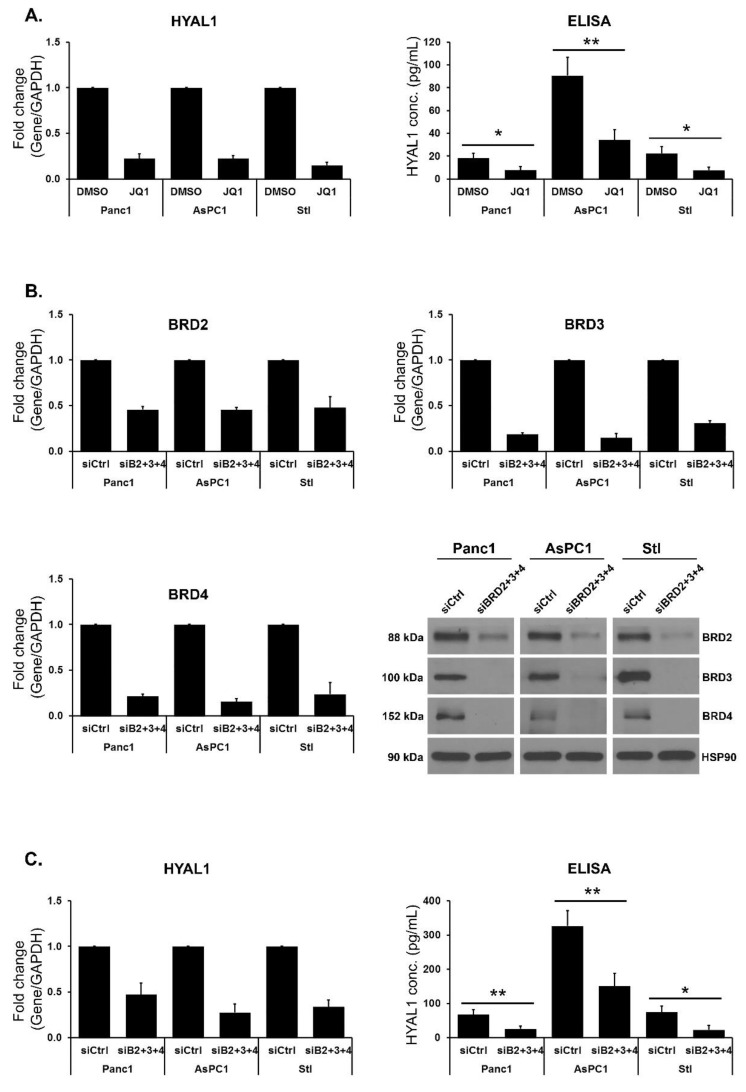
Targeting BET proteins decreases HYAL1 expression. (**A**) PDAC cell lines Panc1 and AsPC1 and the immortalized stellate (Stl) cell line were treated with DMSO or JQ1 for 24 h, and the effect on HYAL1 mRNA expression was determined by qRT-PCR. HYAL1 protein concentration in cell culture media was measured by HYAL1 ELISA after 72 h of treatment with JQ1 or control (DMSO). Mean ± SD. (**B**) Panc1, AsPC1 and Stl cell lines were transfected with control siRNA or with a combination of siRNAs against BRD2, BRD3, and BRD4 (siB2+3+4). The efficiency of BRD2, BRD3, and BRD4 knockdown was determined at the mRNA level after 48 h of transfection by qRT-PCR (the mean ± SD) and at the protein level by western blotting after 72 h of transfection. (**C**) The effect of siB2+3+4 on HYAL1 expression was determined by qRT-PCR and by ELISA after 96 h of transfection. Mean ± SD. The western blot results are representative of three independent experiments. The qRT-PCR and ELISA results are the mean of three independent experiments. Statistical analysis of ELISA results was done by Student’s *t*-test, * *p* < 0.05; ** *p* < 0.01.

Targeting BET proteins decreases HYAL1 expression. Since we previously showed that BET inhibitors can decrease fibrosis [27], we evaluated the effects of targeting BET proteins on HYAL expression in pancreatic cancer cells (Panc1 and AsPC1) and in the pancreatic stellate cell line. The cells were treated with the well-established BET inhibitor JQ1, and the effect on HYAL1, HYAL2, and HYAL3 was determined. JQ1 consistently decreased HYAL1 expression at mRNA and protein levels in Panc1, AsPC1 and the stellate cell line (Figure 2A). In contrast, JQ1 had variable effects on HYAL2 and HYAL3 mRNA expression (Supplementary File: Figure S2). As JQ1 treatment qualitatively recapitulates the phenotype of siRNA-mediated knockdown of BET protein [40,41], we evaluated the effects of co-knockdown of BRD2, BRD3, and BRD4 with siRNAs on HYAL expression. The efficiency of the knockdown of BRD2, BRD3, and BRD4 was confirmed with RT-qPCR and western blotting (Figure 2B). Consistent with our findings with JQ1 treatment, siRNA-mediated co-knockdown of the BET proteins significantly decreased HYAL1 mRNA and protein concentration in all three cell lines (Figure 2C). In contrast, co-knockdown of BRD2, BRD3 and BRD4 had minimal to no effects on HYAL2 and HYAL3 mRNA levels (Supplementary File: Figure S2).

**Figure 3:**
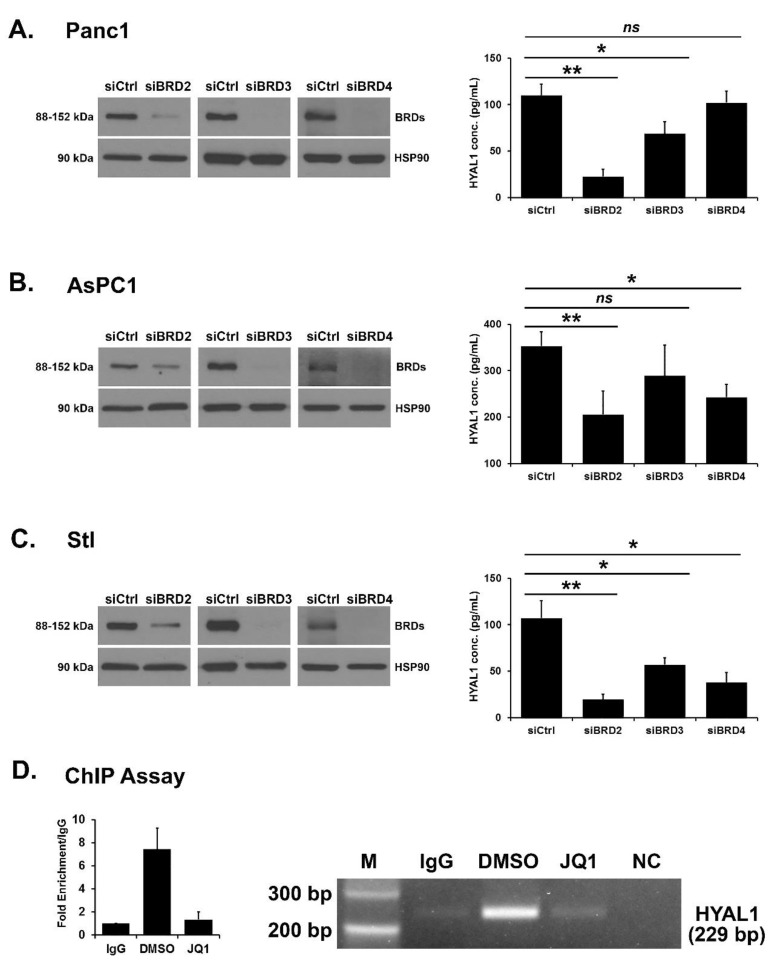
Regulation of HYAL1 expression by BRD2, BRD3 and BRD4. (**A**–**C**) Panc1 (*A*), AsPC1 (*B*), and stellate (Stl, *C*) cells were transfected with control siRNA or individual siRNAs against BRD2 (siB2), BRD3 (siB3), and BRD4 (siB4). The efficiency of BRD2, BRD3, and BRD4 knockdown was determined at the protein level by Western blotting after 72 h of transfection and the effect on HYAL1 protein concentration was determined with ELISA after 96 h of transfection. Mean ± SD. (**D**) AsPC1 cell line was with the BET inhibitor JQ1 (1 μM) overnight. The cells were subjected to ChIP analysis using an anti-BRD2 antibody, and an isotype-matched IgG was used as a negative control. The association with the HYAL1 gene promoter was quantified by qPCR and 2% agarose gel electrophoresis. The qPCR results are the mean of two independent experiments. Mean ± SD. The western blot results are representative of three independent experiments, and ELISA results are the mean of three independent experiments. Statistical analysis of ELISA results was done by Student’s *t*-test, ns *p* > 0.05; * *p* < 0.05; ** *p* < 0.01. NC, no template control.

Regulation of HYAL1 expression by BRD2, BRD3 and BRD4. We next evaluated the role of the different BRD proteins in regulating HYAL1 expression by transfecting Panc1, AsPC1 and stellate cells with individual siRNAs against BRD2, BRD3, or BRD4. The efficiency of knockdown was confirmed by Western blotting, and the effect on HYAL1 protein levels was determined with ELISA (Figure 3A–C). Targeting BRD2 decreased HYAL1 protein concentration most efficiently and consistently in all three cell lines. In contrast, BRD3 and BRD4 knockdown showed modest effects on HYAL1 protein concentration.

BRD2 protein binds to the HYAL1 promoter. Given our results that targeting BET proteins, particularly BRD2, decreases HYAL1 levels, we evaluated BRD2 regulation of HYAL1 mRNA expression. Previous studies have shown that BRD2 regulates gene expression by binding to their promoters [42,43]. AsPC1 cells were treated with DMSO or JQ1, and the effect on BRD2 binding to the HYAL1 promoter was determined by ChIP assay. Compared to control IgG, there was ~6-fold enrichment of the HYAL1 promoter with the BRD2 antibody in the ChIP assay. Importantly, JQ1 treatment decreased the binding of BRD2 protein to the HYAL1 promoter by ~5-fold (Figure 3D). These results demonstrate that BRD2 directly regulates HYAL1 gene expression by binding to its promoter.

**Figure 4:**
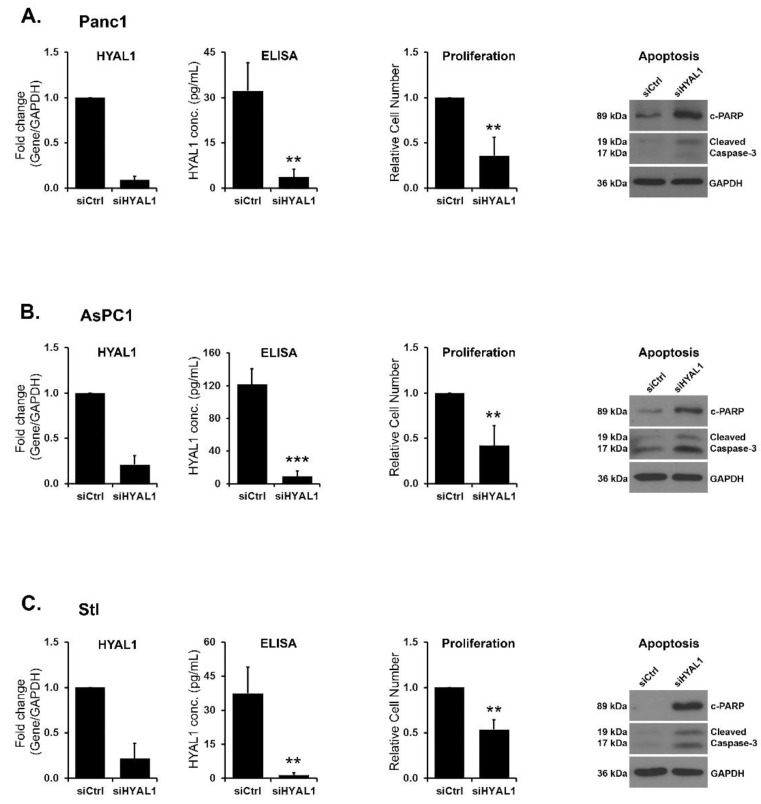
HYAL1 knockdown decreases proliferation and induces apoptosis. (**A**–**C**) Panc1 (*A*), AsPC1 (*B*), and stellate (Stl, *C*) cells were transfected with control siRNA or siRNAs against HYAL1. The effect on HYAL1 mRNA was determined after 48 h of transfection by qRT-PCR, and the effect of HYAL1 protein was determined by ELISA after 96 h of transfection. Mean ± SD. The effect on cell proliferation was analyzed with WST-1 assay (mean ± SD), and the effect on apoptosis was analyzed with western blotting for cleaved PARP and cleaved caspase-3 after 48 h of transfection. The western blot results are representative of three independent experiments. The qRT-PCR and ELISA assay results are the mean of three independent experiments. Statistical analysis of ELISA results was done by Student’s *t*-test and relative proliferation by Mann–Whitney U test, ** *p* < 0.01 *** *p* < 0.001.

HYAL1 knockdown decreases proliferation and induces apoptosis. To investigate the functional role of HYAL1, we downregulated HYAL1 in Panc1, AsPC1 and stellate cells and evaluated the effect on proliferation (Figure 4). HYAL1 siRNA decreased HYAL1 mRNA and protein levels and significantly decreased proliferation (Figure 4). The effect of HYAL1 silencing on cell proliferation was confirmed using a different HYAL1 siRNA (Supplementary File: Figure S3). In contrast, siRNA targeting HYAL2 did not affect proliferation (Supplementary File: Figure S4), and siRNA targeting HYAL3 decreased proliferation only in AsPC1 cells (Supplementary File: Figure S5). We also evaluated the effects of targeting HYAL1 on apoptosis. HYAL1 siRNA decreased HYAL1 protein expression and increased caspase-3 and PAPR cleavage (Figure 4A–C), indicating that HYAL1 knockdown results in increased apoptosis.

**Figure 5:**
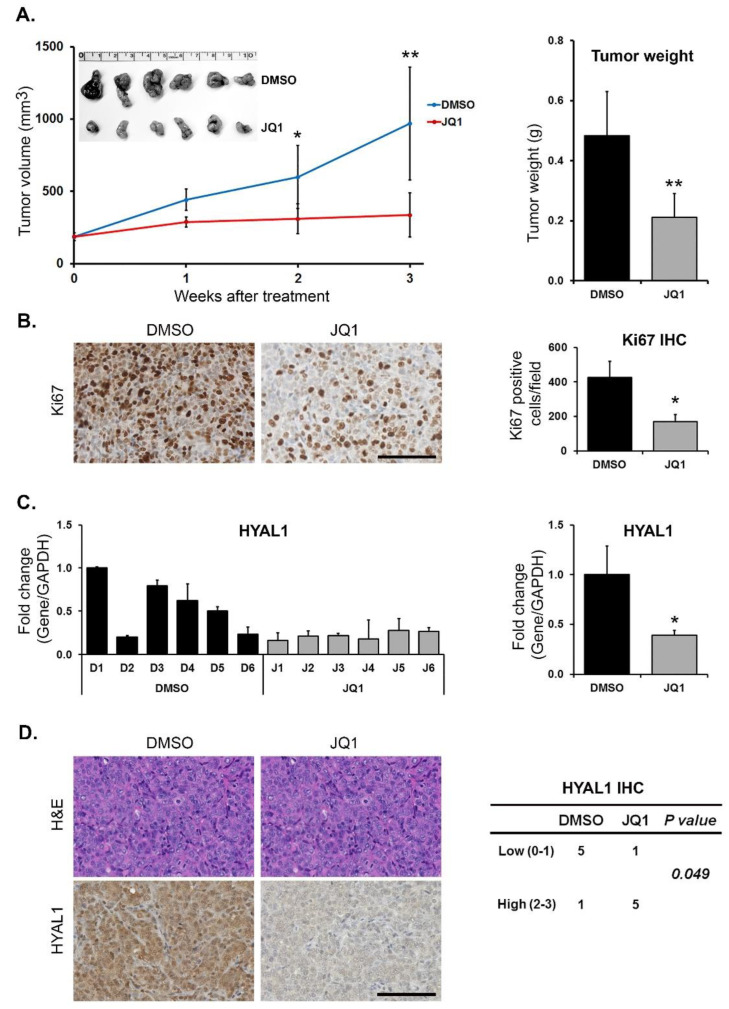
Targeting BET proteins decreases HYAL1 expression and proliferation in vivo. Mice with established Panc1 tumors growing subcutaneously were treated with DMSO (Vehicle) or JQ1 (50 mg/kg, daily for three weeks). (**A**) Changes in tumor volume in DMSO– and JQ1–treated groups, with data shown as mean tumor volume ± SD. (**B**) The tumor sections were stained with Ki67 antibody to evaluate for cell proliferation and quantified as described in Methods. (**C**) The effect on HYAL1 mRNA levels in individual tumors was determined by qRT–PCR and the mean (±SD). HYAL1 mRNA expression in DMSO– and JQ1–treated groups were also analyzed, with data shown as average fold change ± SD. (**D**) The tumor sections were stained with H&E and for HYAL1 expression, and the relative expression was quantified as described in Methods. Scale bar = 100 μm. Statistical analysis of tumor volume, tumor weight and IHC staining was done by Student’s *t*-test and relative mRNA expression by Mann–Whitney U test, * *p* < 0.05; ** *p* < 0.01.

Targeting BET proteins decreases HYAL1 expression and proliferation in vivo. Finally, we evaluated the effects of JQ1 on HYAL expression in vivo. Established Panc1 tumors were treated with JQ1 daily, and the effect on tumor growth was monitored. JQ1 reduced tumor growth, with a significant decrease in tumor weight at the end of the experiment (Figure 5A). JQ1 also significantly decreased proliferation, as determined by Ki67 staining (Figure 5B). There was a significant decrease in HYAL1 mRNA levels in JQ1-treated tumors compared to control tumors (Figure 5C), but not in HYAL2 and HYAL3 mRNA levels between DMSO- and JQ1-treated tumors (Supplementary File: Figure S6). The decrease in HYAL1 expression in JQ1-treated tumors was also confirmed at the protein level by IHC staining for HYAL1 (Figure 5D).

## 4. Discussion

Multiple studies have shown overexpression of HA and HYALs in various cancers, including PDAC [44]. HA overproduction and degradation are essential for PDAC tumor progression [45]. The HA molecule has a unique ability to imbibe a significant amount of water. Thus, high levels of HA increase interstitial pressure and impair drug entry in PDAC tumor cells, and consequently promote chemoresistance in tumors [7]. High levels of HYALs increase the low molecular weight HA during enzymatic degradation of high molecular weight HA [46]. The low molecular weight HA acts as a signaling molecule by interacting with specific cell surface receptors, such as the receptor for HA-mediated motility (RHAMM) and CD44, to modulate a variety of cellular processes [47]. In this study, we show that HYAL1, HYAL2, and HYAL3 are expressed in human PDAC tumors and in PDAC cell lines. We also show that BET inhibition decreases HYAL1 expression without significantly affecting the levels of HYAL2 and HYAL3.

HYAL1 gene transcription is dysregulated by epigenetic alterations, such as promoter methylation and histone acetylation [17]. In recent years, epigenetic readers, especially BET proteins, have become attractive targets for cancer therapeutics [21]. BET inhibitors are also being evaluated in combination with various therapies. For example, JQ1 has been reported to both radiosensitize PDAC cells and enhance the efficacy of gemcitabine, the standard first-line chemotherapy drug for PDAC [48,49,50]. In this study, we show that HYAL1 expression is primarily regulated by BRD2. In contrast, we previously showed that the expression of collagen in stellate cells was primarily regulated by BRD4 [27]. A number of studies have now shown that BET proteins have both overlapping and distinct functions in gene expression [51,52]. For example, BRD2 enhances epithelial-mesenchymal transition (EMT) in breast cancer cells, while BRD3 and BRD4 repress EMT [53]. BRD2, but not BRD3 or BRD4, regulates interleukin 8 production during keratinocyte inflammatory response [54]. Also, isoform switching of BRD2 and BRD4 regulates Smad2-dependent lineage specification of pluripotent stem cells [55].

While HYAL1 overexpression can induce migration, invasion, and metastasis in different cancer models [56,57,58], HYAL1 can also function as a tumor suppressor in some cancers [59]. For example, in prostate cancer cell lines, HYAL1 functions as an oncogene or a tumor suppressor, depending on the HYAL1 levels [60]. However, in PDAC cells, our results show a decreased proliferation in cells expressing high as well as low levels of HYAL1 upon HYAL1 silencing. In our study of five PDAC cell lines, we found that AsPC1 expresses the highest level of HYAL1 mRNA. Our findings are in agreement with a previous study demonstrating that HYAL1 mRNA is variably expressed in a panel of nine PDAC cell lines [17]. Four PDAC cell lines (AsPC1, BxPC3, CFPAC1 and NORP1) showed particularly high levels of HYAL1 mRNA [17]. We have also found that HYAL1 knockdown induces apoptosis in PDAC cells. Our findings are in contrast to the findings in prostate cancer, where HYAL1 overexpression was shown to induce apoptosis [60]. These results suggest that the role of HYAL1 may be dependent on the tumor type.

## 5. Conclusions

We show for the first time that BET inhibitors downregulate HYAL1 expression and that HYAL1 expression is primarily regulated by BRD2. We also show that HYAL1 downregulation decreases proliferation and enhances apoptosis. Thus, our study provides novel insights into the regulation and functional involvement of HYAL1 in PDAC and highlights HYAL1 as a promising therapeutic target for PDAC.

## Data Availability

Uncropped and unprocessed images of western blots have been uploaded to the MDPI as part of this submission.

## Supplementary Materials

The following supporting information can be downloaded at: https://www.mdpi.com/article/10.3390/cells12111490/s1, Figure S1: Expression of Hyaluronidase-2 (HYAL2) and Hyaluronidase-3 (HYAL3) in PDAC specimens and cell lines; Figure S2: Effect of targeting BET proteins on HYAL2 and HYAL3 expression; Figure S3: HYAL1 knockdown decreases cell proliferation; Figure S4: HYAL2 knockdown does not decrease cell pro-liferation; Figure S5: Effect of HYAL3 knockdown on proliferation; Figure S6: Effect of JQ1 on HYAL2 and HYAL3 expression in vivo.

## Abbreviations

BET	Bromodomain and Extra-Terminal Domain
BRD2	Bromodomain-containing protein 2
BRD3	Bromodomain-containing protein 3
BRD4	Bromodomain-containing protein 4
ChIP	Chromatin immunoprecipitation
DMSO	Dimethyl sulfoxide
ELISA	Enzyme-linked immunosorbent assay
HA	Hyaluronan (Hyaluronic acid)
HABP	Hyaluronic acid binding protein
HASs	Hyaluronan synthases
HMW-HA	High Molecular Weight Hyaluronan (Hyaluronic acid)
HYAL1	Hyaluronidase-1
HYAL2	Hyaluronidase-2
HYAL3	Hyaluronidase-3
IHC	Immunohistochemical staining
LMW-HA	Low Molecular Weight Hyaluronan (Hyaluronic acid)
PDAC	Pancreatic ductal adenocarcinoma
qRT-PCR	Quantitative reverse transcription PCR
Stl	Human pancreatic stellate cell line

## References

[1] Rahib L., Smith B.D., Aizenberg R., Rosenzweig A.B., Fleshman J.M., Matrisian L.M. (2014). Projecting cancer incidence and deaths to 2030: The unexpected burden of thyroid, liver, and pancreas cancers in the United States. Cancer Res..

[2] Rucki A.A., Zheng L. (2014). Pancreatic cancer stroma: Understanding biology leads to new therapeutic strategies. World J. Gastroenterol..

[3] Rucki A.A., Foley K., Zhang P., Xiao Q., Kleponis J., Wu A.A., Sharma R., Mo G., Liu A., Van Eyk J. (2017). Heterogeneous Stromal Signaling within the Tumor Microenvironment Controls the Metastasis of Pancreatic Cancer. Cancer Res..

[4] Thomas D., Radhakrishnan P. (2019). Tumor-stromal crosstalk in pancreatic cancer and tissue fibrosis. Mol. Cancer.

[5] Itano N., Zhuo L., Kimata K. (2008). Impact of the hyaluronan-rich tumor microenvironment on cancer initiation and progression. Cancer Sci..

[6] Tammi R.H., Kultti A., Kosma V.M., Pirinen R., Auvinen P., Tammi M.I. (2008). Hyaluronan in human tumors: Pathobiological and prognostic messages from cell-associated and stromal hyaluronan. Semin. Cancer Biol..

[7] DuFort C.C., DelGiorno K.E., Hingorani S.R. (2016). Mounting Pressure in the Microenvironment: Fluids, Solids, and Cells in Pancreatic Ductal Adenocarcinoma. Gastroenterology.

[8] Hamester F., Sturken C., Legler K., Eylmann K., Moller K., Rossberg M., Gorzelanny C., Bauer A.T., Windhorst S., Schmalfeldt B. (2022). Key Role of Hyaluronan Metabolism for the Development of Brain Metastases in Triple-Negative Breast Cancer. Cells.

[9] Schmaus A., Klusmeier S., Rothley M., Dimmler A., Sipos B., Faller G., Thiele W., Allgayer H., Hohenberger P., Post S. (2014). Accumulation of small hyaluronan oligosaccharides in tumour interstitial fluid correlates with lymphatic invasion and lymph node metastasis. Br. J. Cancer.

[10] Tavianatou A.G., Caon I., Franchi M., Piperigkou Z., Galesso D., Karamanos N.K. (2019). Hyaluronan: Molecular size-dependent signaling and biological functions in inflammation and cancer. FEBS J..

[11] Caon I., Bartolini B., Parnigoni A., Carava E., Moretto P., Viola M., Karousou E., Vigetti D., Passi A. (2020). Revisiting the hallmarks of cancer: The role of hyaluronan. Semin. Cancer Biol..

[12] Riecks J., Parnigoni A., Gyorffy B., Kiesel L., Passi A., Vigetti D., Gotte M. (2022). The hyaluronan-related genes HAS2, HYAL1-4, PH20 and HYALP1 are associated with prognosis, cell viability and spheroid formation capacity in ovarian cancer. J. Cancer Res. Clin. Oncol..

[13] McAtee C.O., Booth C., Elowsky C., Zhao L., Payne J., Fangman T., Caplan S., Henry M.D., Simpson M.A. (2019). Prostate tumor cell exosomes containing hyaluronidase Hyal1 stimulate prostate stromal cell motility by engagement of FAK-mediated integrin signaling. Matrix Biol..

[14] Lokeshwar V.B., Rubinowicz D., Schroeder G.L., Forgacs E., Minna J.D., Block N.L., Nadji M., Lokeshwar B.L. (2001). Stromal and epithelial expression of tumor markers hyaluronic acid and HYAL1 hyaluronidase in prostate cancer. J. Biol. Chem..

[15] Stern R. (2004). Hyaluronan catabolism: A new metabolic pathway. Eur. J. Cell Biol..

[16] Csoka A.B., Frost G.I., Stern R. (2001). The six hyaluronidase-like genes in the human and mouse genomes. Matrix Biol..

[17] Kohi S., Sato N., Cheng X.B., Koga A., Hirata K. (2016). Increased Expression of HYAL1 in Pancreatic Ductal Adenocarcinoma. Pancreas.

[18] Cheng X.B., Sato N., Kohi S., Yamaguchi K. (2013). Prognostic impact of hyaluronan and its regulators in pancreatic ductal adenocarcinoma. PLoS ONE.

[19] Shankar D., Merchand-Reyes G., Buteyn N.J., Santhanam R., Fang H., Kumar K., Mo X., Ganesan L.P., Jarjour W., Butchar J.P. (2023). Inhibition of BET Proteins Regulates Fcgamma Receptor Function and Reduces Inflammation in Rheumatoid Arthritis. Int. J. Mol. Sci..

[20] Kanojia D., Panek W.K., Cordero A., Fares J., Xiao A., Savchuk S., Kumar K., Xiao T., Pituch K.C., Miska J. (2020). BET inhibition increases betaIII-tubulin expression and sensitizes metastatic breast cancer in the brain to vinorelbine. Sci. Transl. Med..

[21] Donati B., Lorenzini E., Ciarrocchi A. (2018). BRD4 and Cancer: Going beyond transcriptional regulation. Mol. Cancer.

[22] Dhalluin C., Carlson J.E., Zeng L., He C., Aggarwal A.K., Zhou M.M. (1999). Structure and ligand of a histone acetyltransferase bromodomain. Nature.

[23] Xie F., Huang M., Lin X., Liu C., Liu Z., Meng F., Wang C., Huang Q. (2018). The BET inhibitor I-BET762 inhibits pancreatic ductal adenocarcinoma cell proliferation and enhances the therapeutic effect of gemcitabine. Sci. Rep..

[24] Miller A.L., Garcia P.L., Yoon K.J. (2020). Developing effective combination therapy for pancreatic cancer: An overview. Pharmacol. Res..

[25] Garcia P.L., Miller A.L., Kreitzburg K.M., Council L.N., Gamblin T.L., Christein J.D., Heslin M.J., Arnoletti J.P., Richardson J.H., Chen D. (2016). The BET bromodomain inhibitor JQ1 suppresses growth of pancreatic ductal adenocarcinoma in patient-derived xenograft models. Oncogene.

[26] Doroshow D.B., Eder J.P., LoRusso P.M. (2017). BET inhibitors: A novel epigenetic approach. Ann. Oncol..

[27] Kumar K., DeCant B.T., Grippo P.J., Hwang R.F., Bentrem D.J., Ebine K., Munshi H.G. (2017). BET inhibitors block pancreatic stellate cell collagen I production and attenuate fibrosis in vivo. JCI Insight.

[28] Sherman M.H., Yu R.T., Tseng T.W., Sousa C.M., Liu S., Truitt M.L., He N., Ding N., Liddle C., Atkins A.R. (2017). Stromal cues regulate the pancreatic cancer epigenome and metabolome. Proc. Natl. Acad. Sci. USA.

[29] Hwang R.F., Moore T., Arumugam T., Ramachandran V., Amos K.D., Rivera A., Ji B., Evans D.B., Logsdon C.D. (2008). Cancer-associated stromal fibroblasts promote pancreatic tumor progression. Cancer Res..

[30] Shields M.A., Ebine K., Sahai V., Kumar K., Siddiqui K., Hwang R.F., Grippo P.J., Munshi H.G. (2013). Snail cooperates with KrasG12D to promote pancreatic fibrosis. Mol. Cancer Res..

[31] Pham T.N.D., Kumar K., DeCant B.T., Shang M., Munshi S.Z., Matsangou M., Ebine K., Munshi H.G. (2019). Induction of MNK Kinase-dependent eIF4E Phosphorylation by Inhibitors Targeting BET Proteins Limits Efficacy of BET Inhibitors. Mol. Cancer Ther..

[32] Sahai V., Kumar K., Knab L.M., Chow C.R., Raza S.S., Bentrem D.J., Ebine K., Munshi H.G. (2014). BET bromodomain inhibitors block growth of pancreatic cancer cells in three-dimensional collagen. Mol. Cancer Ther..

[33] Kumar K., Raza S.S., Knab L.M., Chow C.R., Kwok B., Bentrem D.J., Popovic R., Ebine K., Licht J.D., Munshi H.G. (2015). GLI2-dependent c-MYC upregulation mediates resistance of pancreatic cancer cells to the BET bromodomain inhibitor JQ1. Sci. Rep..

[34] Kumar K., Chow C.R., Ebine K., Arslan A.D., Kwok B., Bentrem D.J., Eckerdt F.D., Platanias L.C., Munshi H.G. (2016). Differential Regulation of ZEB1 and EMT by MAPK-Interacting Protein Kinases (MNK) and eIF4E in Pancreatic Cancer. Mol. Cancer Res..

[35] Lokeshwar V.B., Gomez P., Kramer M., Knapp J., McCornack M.A., Lopez L.E., Fregien N., Dhir N., Scherer S., Klumpp D.J. (2008). Epigenetic regulation of HYAL-1 hyaluronidase expression. identification of HYAL-1 promoter. J. Biol. Chem..

[36] Kumar K., Wigfield S., Gee H.E., Devlin C.M., Singleton D., Li J.L., Buffa F., Huffman M., Sinn A.L., Silver J. (2013). Dichloroacetate reverses the hypoxic adaptation to bevacizumab and enhances its antitumor effects in mouse xenografts. J. Mol. Med. (Berl).

[37] Stern R. (2003). Devising a pathway for hyaluronan catabolism: Are we there yet?. Glycobiology.

[38] Lepperdinger G., Mullegger J., Kreil G. (2001). Hyal2--less active, but more versatile?. Matrix Biol..

[39] Harada H., Takahashi M. (2007). CD44-dependent intracellular and extracellular catabolism of hyaluronic acid by hyaluronidase-1 and -2. J. Biol. Chem..

[40] Filippakopoulos P., Qi J., Picaud S., Shen Y., Smith W.B., Fedorov O., Morse E.M., Keates T., Hickman T.T., Felletar I. (2010). Selective inhibition of BET bromodomains. Nature.

[41] Belkina A.C., Nikolajczyk B.S., Denis G.V. (2013). BET protein function is required for inflammation: Brd2 genetic disruption and BET inhibitor JQ1 impair mouse macrophage inflammatory responses. J. Immunol..

[42] Mota de Sa P., Richard A.J., Stephens J. (2019). BET inhibition by JQ1 produces divergent transcriptional regulation of SOCS genes in adipocytes. Endocrinology.

[43] Sinha A., Faller D.V., Denis G.V. (2005). Bromodomain analysis of Brd2-dependent transcriptional activation of cyclin A. Biochem. J..

[44] McAtee C.O., Berkebile A.R., Elowsky C.G., Fangman T., Barycki J.J., Wahl J.K., Khalimonchuk O., Naslavsky N., Caplan S., Simpson M.A. (2015). Hyaluronidase Hyal1 Increases Tumor Cell Proliferation and Motility through Accelerated Vesicle Trafficking. J. Biol. Chem..

[45] Sato N., Kohi S., Hirata K., Goggins M. (2016). Role of hyaluronan in pancreatic cancer biology and therapy: Once again in the spotlight. Cancer Sci..

[46] Wu M., Cao M., He Y., Liu Y., Yang C., Du Y., Wang W., Gao F. (2015). A novel role of low molecular weight hyaluronan in breast cancer metastasis. FASEB J..

[47] Misra S., Hascall V.C., Markwald R.R., Ghatak S. (2015). Interactions between Hyaluronan and Its Receptors (CD44, RHAMM) Regulate the Activities of Inflammation and Cancer. Front. Immunol..

[48] Garcia P.L., Miller A.L., Zeng L., van Waardenburg R., Yang E.S., Yoon K.J. (2022). The BET Inhibitor JQ1 Potentiates the Anticlonogenic Effect of Radiation in Pancreatic Cancer Cells. Front. Oncol..

[49] Miller A.L., Garcia P.L., Fehling S.C., Gamblin T.L., Vance R.B., Council L.N., Chen D., Yang E.S., van Waardenburg R., Yoon K.J. (2021). The BET Inhibitor JQ1 Augments the Antitumor Efficacy of Gemcitabine in Preclinical Models of Pancreatic Cancer. Cancers.

[50] Yamamoto K., Tateishi K., Kudo Y., Hoshikawa M., Tanaka M., Nakatsuka T., Fujiwara H., Miyabayashi K., Takahashi R., Tanaka Y. (2016). Stromal remodeling by the BET bromodomain inhibitor JQ1 suppresses the progression of human pancreatic cancer. Oncotarget.

[51] Cheung K.L., Zhang F., Jaganathan A., Sharma R., Zhang Q., Konuma T., Shen T., Lee J.Y., Ren C., Chen C.H. (2017). Distinct Roles of Brd2 and Brd4 in Potentiating the Transcriptional Program for Th17 Cell Differentiation. Mol. Cell.

[52] Roberts T.C., Etxaniz U., Dall’Agnese A., Wu S.Y., Chiang C.M., Brennan P.E., Wood M.J.A., Puri P.L. (2017). BRD3 and BRD4 BET Bromodomain Proteins Differentially Regulate Skeletal Myogenesis. Sci. Rep..

[53] Andrieu G.P., Denis G.V. (2018). BET Proteins Exhibit Transcriptional and Functional Opposition in the Epithelial-to-Mesenchymal Transition. Mol. Cancer Res..

[54] Slivka P.F., Hsieh C.L., Lipovsky A., Pratt S.D., Locklear J., Namovic M.T., McDonald H.A., Wetter J., Edelmayer R., Hu M. (2019). Small Molecule and Pooled CRISPR Screens Investigating IL17 Signaling Identify BRD2 as a Novel Contributor to Keratinocyte Inflammatory Responses. ACS Chem. Biol..

[55] Fernandez-Alonso R., Davidson L., Hukelmann J., Zengerle M., Prescott A.R., Lamond A., Ciulli A., Sapkota G.P., Findlay G.M. (2017). Brd4-Brd2 isoform switching coordinates pluripotent exit and Smad2-dependent lineage specification. EMBO Rep..

[56] Witzel I., Marx A.K., Muller V., Wikman H., Matschke J., Schumacher U., Sturken C., Prehm P., Laakmann E., Schmalfeldt B. (2017). Role of HYAL1 expression in primary breast cancer in the formation of brain metastases. Breast Cancer Res. Treat..

[57] McAtee C.O., Barycki J.J., Simpson M.A. (2014). Emerging roles for hyaluronidase in cancer metastasis and therapy. Adv. Cancer Res..

[58] Kohi S., Sato N., Koga A., Hirata K., Harunari E., Igarashi Y. (2016). Hyaluromycin, a Novel Hyaluronidase Inhibitor, Attenuates Pancreatic Cancer Cell Migration and Proliferation. J. Oncol..

[59] Wang F., Grigorieva E.V., Li J., Senchenko V.N., Pavlova T.V., Anedchenko E.A., Kudryavtseva A.V., Tsimanis A., Angeloni D., Lerman M.I. (2008). HYAL1 and HYAL2 inhibit tumour growth in vivo but not in vitro. PLoS ONE.

[60] Lokeshwar V.B., Cerwinka W.H., Isoyama T., Lokeshwar B.L. (2005). HYAL1 hyaluronidase in prostate cancer: A tumor promoter and suppressor. Cancer Res..

