# Supplementary material for: Targeting BET Proteins Decreases Hyaluronidase-1 in Pancreatic Cancer
Source: Cells. 2023 May 27;12(11):1490. doi: 10.3390/cells12111490 (PMC10253193; doi:10.3390/cells12111490)
Supplement: Supplementary file 1 [file cells-12-01490-s001.zip › cells-2346542-supplementary.pdf]

Supplementary Figure and Supplementary Figure Legends

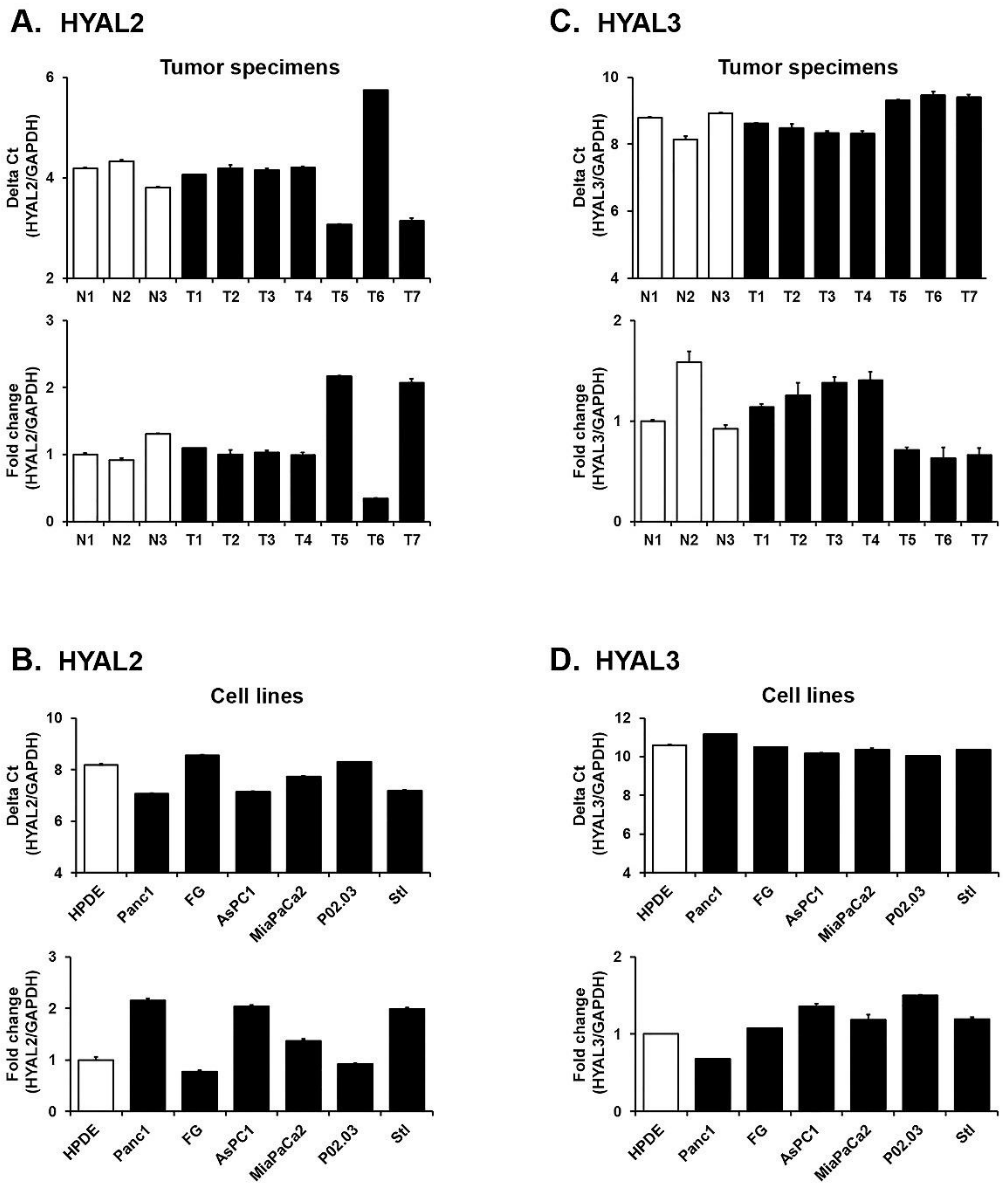

Figure S1. Expression of Hyaluronidase-2 (HYAL2) and Hyaluronidase-3 (HYAL3) in PDAC specimens and cell lines. (A, C) HYAL2 and HYAL3 mRNA levels were analyzed in 3 normal human pancreatic tissue samples and in 7 PDAC specimens. The relative HYAL2 and HYAL3 mRNA levels

were normalized to the corresponding mRNA levels present in normal human pancreatic tissue 1 (N1) for tumor specimens, shown as delta Ct (upper panel) and fold change (lower panel). Mean ± SD. **(B, D)** HYAL1 and HYAL3 mRNA levels were analyzed in immortalized human pancreatic duct epithelial (HPDE) cells, in a panel of 5 human PDAC cell lines and in an immortalized human pancreatic stellate cell line (Stl). The relative mRNA levels were normalized to the corresponding mRNA levels present in HPDE cells, shown as delta Ct (upper panel) and fold change (lower panel). Mean ± SD. The gene expression results are representative of three independent experiments.

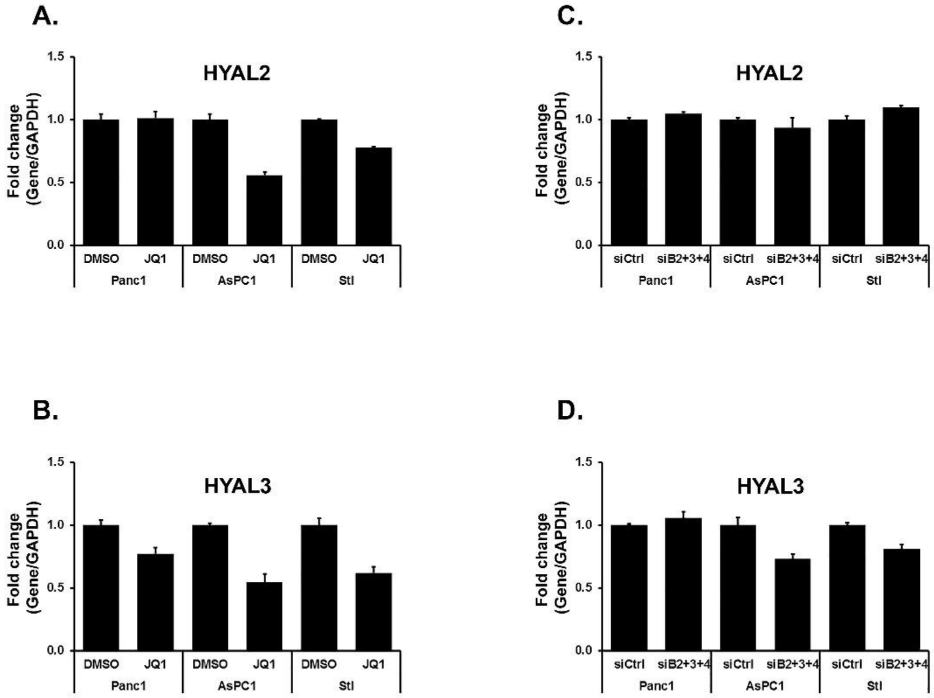

Figure S2. Effect of targeting BET proteins on HYAL2 and HYAL3 expression. (A, B) PDAC cell lines Panc1 and AsPC1, and the immortalized stellate (Stl) cell line, were treated with DMSO or JQ1 for 24 hours. The effects on HYAL2 mRNA and HYAL3 mRNA levels were determined by qRT-PCR (mean ± SD shown). **(C, D)** Panc1, AsPC1 and Stl cell lines were transfected with control siRNA or with a combination of siRNAs against BRD2, BRD3, and BRD4 (siB2+3+4), and the effects on HYAL2 and HYAL3 mRNA levels were determined after 96 hours of transfection by qRT-PCR (mean ± SD shown). The results are representative of three independent experiments.

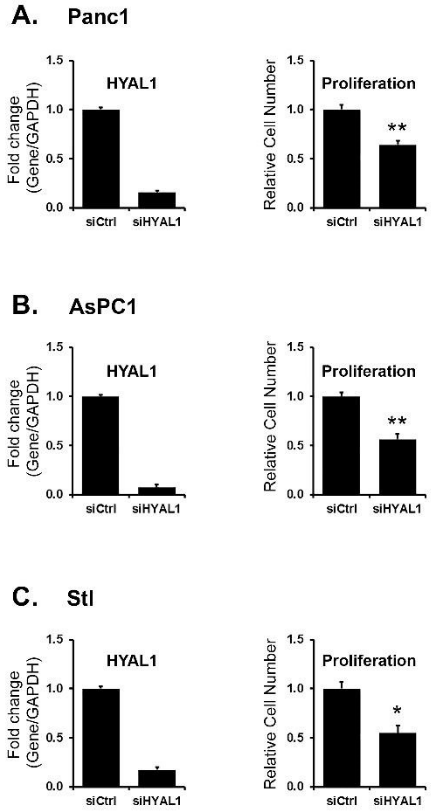

Figure S3. HYAL1 knockdown decreases cell proliferation. (A-C) Panc1 (*A*), AsPC1 (*B*), and stellate (Stl, *C*) cells were transfected with control siRNA or siRNAs against HYAL1. HYAL1 knockdown was determined after 48 hours of transfection by qRT-PCR (mean ± SD shown). The effects of HYAL1 knockdown on cell proliferation were analyzed via WST-1 assay. Mean ± SD. The results are representative of three independent experiments. *p < 0.05; **p < 0.01.

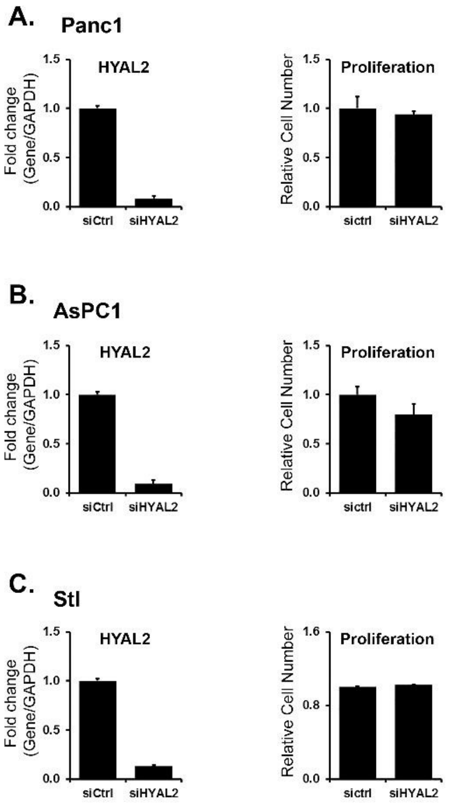

Figure S4. HYAL2 knockdown does not decrease cell proliferation. (A-C) Panc1 (*A*), AsPC1 (*B*) and stellate (Stl, *C*) cells were transfected with control siRNA or siRNAs against HYAL2. HYAL2 knockdown was determined after 48 hours of transfection by qRT-PCR (mean ± SD shown). The effects of HYAL2 knockdown on cell proliferation were analyzed with WST-1 assay. Mean ± SD. The results are representative of three independent experiments.

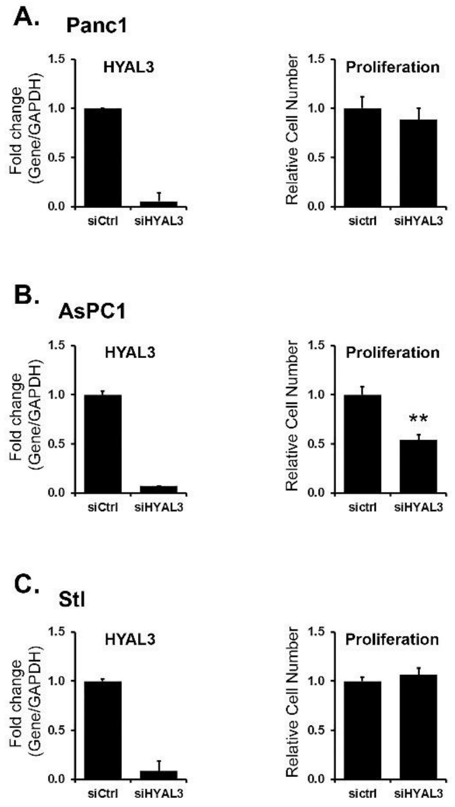

Figure S5. Effect of HYAL3 knockdown on proliferation. (A-C) Panc1 (*A*), AsPC1 (*B*) and stellate (Stl, *C*) cells were transfected with control siRNA or siRNAs against HYAL3. HYAL3 knockdown was determined after 48 hours of transfection by qRT-PCR. The effect of HYAL3 knockdown on cell proliferation was analyzed with WST-1 assay. Mean ± SD. The results are representative of three independent experiments. *p < 0.05; **p < 0.01.

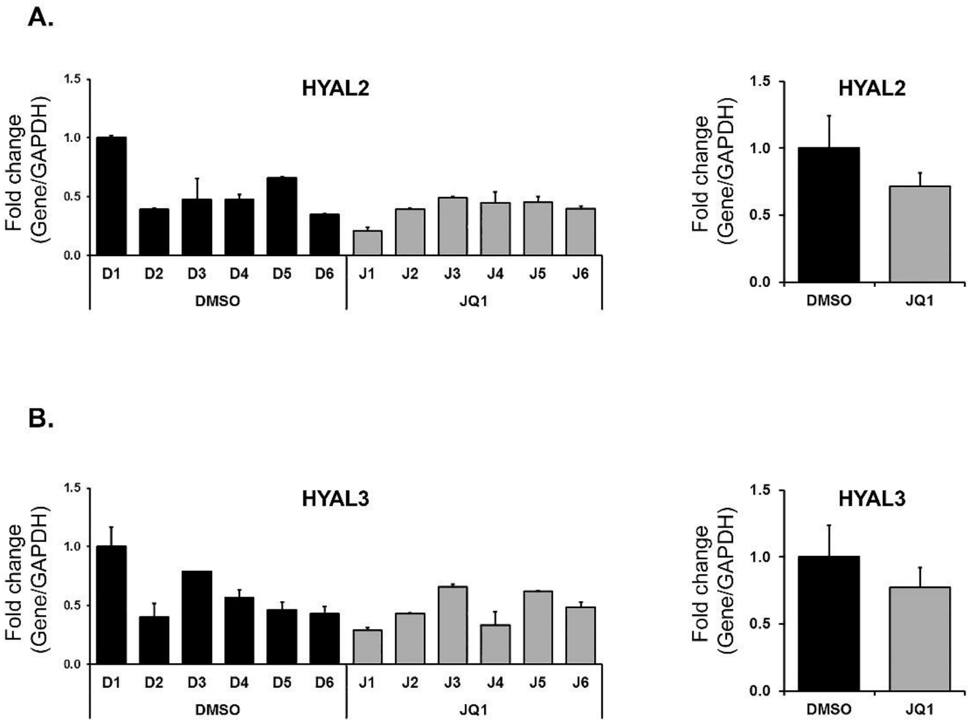

Figure S6. Effect of JQ1 on HYAL2 and HYAL3 expression *in vivo*. Mice with established Panc1 tumors growing subcutaneously were treated with DMSO (Vehicle) or JQ1 (50mg/kg, daily for three

weeks). **(A)** The effect on HYAL2 mRNA levels in individual tumors was determined by qRT-PCR (mean ± SD shown). HYAL2 mRNA expression in DMSO- and JQ1-treated groups was also analyzed, with data shown as average fold change ± SD. **(B)** The effect on HYAL3 mRNA levels in individual tumors was determined by qRT-PCR (mean ± SD shown). HYAL3 mRNA expression in DMSO- and JQ1-treated groups was also analyzed, with data shown as average fold change ± SD.
